# Effects of orally administered cetylated fatty acids on symptoms and functional capacity in patients with knee osteoarthritis: results of a randomized, double-blind, placebo-controlled study

**DOI:** 10.1038/s41430-025-01656-4

**Published:** 2025-08-25

**Authors:** Manana Zodeleva, Nino Pochkhua, Maria Sole Rossato, Eka Arziani

**Affiliations:** 1 JSC “Evex Hospitals” (Caraps Medline), Tbilisi, Georgia; 2 LLC “Altra Vita”, Tbilisi, Georgia; 3 Pharmanutra Spa, Pisa, Italy; 4 LLC “Unica”, Tbilisi, Georgia

**Keywords:** Nutrition, Acute inflammatory arthritis

## Abstract

**Background/Objectives:**

The development and implementation of new treatments for knee osteoarthritis in routine practice remains an unmet need. The aim of this study was to assess the efficacy and safety of a Cetylated Fatty Acids (CFA)-based dietary supplement in patients with knee osteoarthritis (OA), a prevalent and difficult-to-treat condition.

**Subjects/methods:**

60 patients (mean age: 66.0 ± 7.7 years, 85% female) with grade 3–4 knee osteoarthritis and a pain intensity of > 4 cm on the visual analog scale (VAS) were enrolled and randomized in a 1:1 ratio to receive either 1.5 g of oral CFA or a placebo for 60 days. The primary outcome was the change in pain intensity (VAS), secondary outcomes included changes in range of motion (ROM), in the Western Ontario and McMaster Universities Osteoarthritis Index (WOMAC), and the safety profile of the food supplement.

**Results:**

After 60 days of CFA assumption, the mean reduction in pain intensity (VAS) was −1.7 cm (95% CI [−2.0, −1.4]), showing a statistically significant difference compared to placebo (−0.6 cm, 95% CI [−1.0, −0.2]; *p* < 0.005). The mean decrease in the WOMAC total score was also greater in the CFA group (−19.5 vs. −15.8), although the placebo-corrected effect was not statistically significant (−3.7, 95% CI [−8.3, 0.8]; *p* = 0.108). Observed improvements in flexion (3.8° [95% CI: 2.6, 5.0]) and external rotation (2.9° [95% CI: 2.1, 3.8]) were both statistically significant in favor of CFA (*p* ≤ 0.001) compared to placebo. Differences in extension and internal rotation were negligible. The safety profile of the investigational product resulted favorable, considering that only 4 out of 30 patients reported mild adverse events, and none withdrawn from the study due to adverse events.

**Conclusion:**

In patients with knee osteoarthritis, incorporating a CFA oral supplement into the treatment regimen provides superior efficacy in pain relief and range of motion improvement compared to placebo, while maintaining a favorable safety profile.

## Introduction

According to the most recent epidemiological data on the frequency and global burden of osteoarthritis (OA), approximately 607 million people (~7.7% of the world’s population) were affected by OA in 2021, with 46.6 million new cases and 21.3 million disability-adjusted life years [1].

The pathogenesis of OA as a degenerative disease involves cartilage degradation and bone remodeling due to mechanical stress and aging. Moreover, recent research shows that OA affects not only cartilage but also the synovium, subchondral bone, ligaments, and peri-articular muscles, involving both cellular and molecular changes. Inflammation, particularly synovitis, plays a key role in OA progression. Histological findings include synovial hyperplasia, immune cell infiltration, neo angiogenesis, fibrosis, and extracellular matrix remodeling, accompanied by the release of inflammatory cytokines and chemokines (interleukin (IL)-1, IL-6, tumor necrosis factor (TNF)-α, etc.), which drive joint degeneration and cause pain by acting on nociceptors [2–4].

The intrinsic complexity of the disease, along with its clinical and pathophysiological heterogeneity, presents significant treatment challenges. Existing common interventional (e.g., intra-articular injections of hyaluronic acid (HA), glucocorticoids, or platelet-rich plasma) and non-interventional (e.g., non-steroidal anti-inflammatory drugs, acetaminophen, duloxetine, or opioids) pharmacological treatment methods provide limited efficacy and/or short-lived therapeutic benefits, frequently accompanied by adverse effects. Consequently, there is growing interest in exploring and developing non-pharmacological treatment strategies [5–7].

Particular attention has been directed toward the potential benefits of diet and nutrition, given their anti-inflammatory properties. Dietary interventions have been extensively studied in inflammatory rheumatic diseases, such as rheumatoid arthritis and spondyloarthropathies, as well as in OA. Emerging evidence suggests that nutritional factors and dietary intake may positively influence pain, stiffness, function, and other symptoms in patients with OA [8, 9].

Cetylated fatty acids (CFAs) comprise a group of naturally occurring fatty acids from vegetable origin. This group includes compounds such as cetyl myristate, cetyl palmitate, cetyl oleate among others. CFAs have been shown to promote the chondrogenic differentiation of human adipose-derived stem cells by enhancing the expression of chondrogenic markers under chondrogenic induction conditions [10]. In animal models of osteoarthritis, histological analysis revealed a protective effect of fatty acids against cartilage degradation in treated knee joints, with a significant improvement in all articular cartilage parameters, including thickness, volume, and surface integrity [11].

Several clinical studies have demonstrated that CFAs, administered both topically and orally, contribute to relieve pain and improve functional performance in patients with knee and hand OA, tendinopathies (such as shoulder tendon disorders), myofascial pain syndrome of the neck, axial discogenic low back pain and sports injuries [12–21].

The aim of our clinical study was to evaluate the efficacy and safety of the first CFAs-based food supplement approved in Europe, containing a mixture of vegetable-derived fatty acids esterified with cetyl alcohol (Lipocet^®^). This supplement, formulated as an oral gel (Cetilar^®^ ORO, PharmaNutra S.p.A., Italy), was studied in patients with primary knee osteoarthritis.

## Subjects and methods

### Design and participants

This study was approved by the Independent Ethics Committee of LTD “Altra Vita” protocol code 404954699, Independent Ethics Committee of JSC “Evex Hospitals” protocol code 404476205, and Independent Ethics Committee of LCC “Unika” protocol code 406027311, both approved on August 19^th^ 2022 in Tbilisi. The study was conducted in accordance with the guidelines set forth in the Declaration of Helsinki. It was carried out from November 2022 to March 2023 in three hospitals in Tbilisi, Republic of Georgia, in Rheumatology Departments, and registered on ClinicalTrials.gov (NCT06134115).

This was a prospective, randomized, controlled, parallel-group interventional study investigating Cetilar^®^ ORO (PharmaNutra S.p.A., Italy) versus placebo. The trial was designed as a double-blind study, with both patients and investigators responsible for outcome assessment remaining unaware of treatment assignments.

According to predefined selection criteria, adult male and female patients (aged 40–80 years) with primary knee osteoarthritis (classified based on the American College of Rheumatology/European League Against Rheumatism (ACR/EULAR) criteria [22, 23]) and Kellgren-Lawrence grade 3 or 4 disease severity were included in the study. A prerequisite for participation was a patient-reported pain intensity of more than 4 cm on a visual analog scale (VAS). Additionally, patients must not have received intra-articular injections within the 3 months prior to enrollment and/or systemic steroid therapy within the 1 month prior to study participation.

### Objective and measurements

All participants were evaluated before the start of the study treatment (baseline) and 30 and 60 days after treatment initiation. The primary objective of the study is to assess the efficacy of the food supplement in reducing pain severity as assessed by the visual analog scale (VAS). Secondary objectives were the effect on functional capacity, tolerability and compliance. Efficacy outcome assessments were conducted in the outpatient department at the same time points.

Pain severity in the knee was measured using a 10-cm visual analog scale (VAS), ranging from 0 (no pain) to 10 (worst possible pain). Functional impairment was assessed based on the range of motion (ROM), including maximum flexion, extension, internal rotation, and external rotation (measured in degrees using a goniometer). For a comprehensive assessment, the total osteoarthritis index score was evaluated using the Western Ontario and McMaster Universities Osteoarthritis Index (WOMAC) questionnaire. The WOMAC consisted of three subscales: pain (five questions), stiffness (two questions), and physical function (17 questions). Throughout the treatment period, compliance was monitored, and data on concomitant therapy were collected. Additionally, patients were instructed to complete a self-assessment questionnaire and maintain a food habits diary.

To assess tolerability, adverse events were continuously monitored through patient surveys and vital sign measurements, including systolic and diastolic blood pressure and pulse rate. The investigators established the relationship between an AE and study product using the following six categories: Certain, Probable, Possible, Unlikely, Not assessable, Not Related.

### Intervention

The investigational medicinal product, CFA-based oral supplement Cetilar^®^ ORO (PharmaNutra S.p.A., Italy), contained a combination of Cetylated Fatty Acids, primarily cetylated myristic acid and oleic acid. The product was formulated as a ready-to-use food gel in sachets, with each sachet containing 1.5 g of CFAs. The placebo food gel contained all the same ingredients except for the CFA and was identical in appearance and organoleptic properties. The dosage and administration regimen for Cetilar^®^ ORO or placebo was set at one sachet per day for 60 days.

### Statistical analysis

All statistical analyses were performed using SAS software, version 9.4 (SAS Institute Inc.). Continuous data were described using the mean ± standard deviation (SD) and 95% confidence interval (CI), while categorical data were presented as counts and percentages (%). Missing data imputation methods were not used.

The primary efficacy endpoint (VAS) and other efficacy parameters (ROM, WOMAC) were analyzed sequentially by comparing the degree of change between groups 60 days after treatment initiation. Efficacy endpoints were also evaluated after 30 days of treatment. Pairwise comparisons were conducted using a two-sided t-test, with a *p*-value < 0.05 considered statistically significant. Additionally, a sensitivity analysis for outliers was conducted using the 2-sigma method.

## Results

**Fig. 1:**
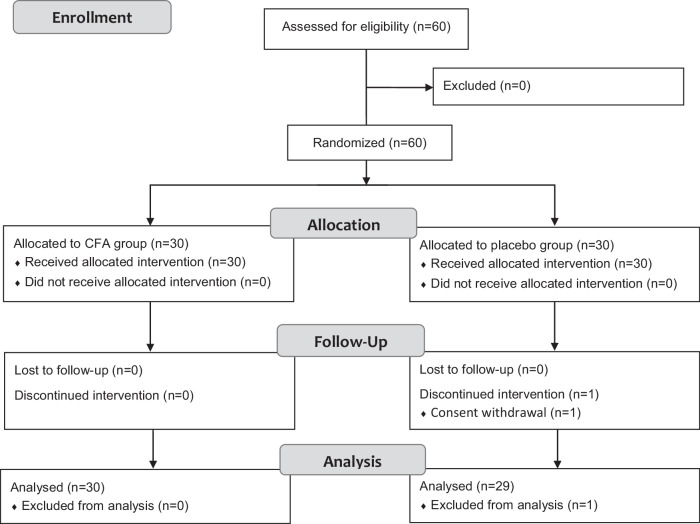
CONSORT flow diagram for the CFA group versus placebo group.

A total of 60 participants with knee osteoarthritis were included in the study; 30 were randomized to the CFA group and 30 to the placebo group (Fig. 1). One patient in the placebo group prematurely withdrew from the study after 30 days of treatment for reasons not associated with the use of study food supplement.

Patients ranged in age from 43 to 78 years (mean 66.0), with the majority being female (85.0%) and having moderate (Grade 3) osteoarthritis (91.7%). The baseline pain intensity (VAS score) in the index knee ranged from 5 to 9 cm, with a mean of 6.6 cm. The demographic and other baseline characteristics were comparable between the two study groups (Table 1).

**Table 1:** Demographic and other baseline characteristics.

	CFA (*N* = 30)	Placebo (*N* = 30)	Total (*N* = 60)
Age (years), mean ± SD (min-max)	67.1 ± 7.6 (43–78)	64.8 ± 7.8 (49–77)	66.0 ± 7.7 (43–78)
Sex			
Male, *n* (%)	3 (10.0)	6 (20.0)	9 (15.0)
Female, *n* (%)	27 (90.0)	24 (80.0)	51 (85.0)
Kellgren-Lawrence classification			
Grade 3 (moderate), *n* (%)	26 (86.7)	29 (96.7)	55 (91.7)
Grade 4 (severe), *n* (%)	4 (13.3)	1 (3.3)	5 (8.3)
Baseline score			
VAS score (cm), mean ± SD	6.56 ± 1.2	6.58 ± 1.1	6.57 ± 1.1
ROM (degrees),			
Flexion, mean ± SD	125.5 ± 3.3	126.1 ± 2.7	125.8 ± 3.0
Extension, mean ± SD	4.2 ± 2.1	4.3 ± 2.0	4.2 ± 2.0
External rotation, mean ± SD	24.6 ± 2.8	25.3 ± 1.8	24.9 ± 2.4
Internal rotation, mean ± SD	7.0 ± 1.1	6.9 ± 0.9	6.9 ± 1.0
WOMAC			
Total score, mean ± SD	56.9 ± 8.7	57.8 ± 12.7	57.4 ± 10.8
Pain, mean ± SD	10.4 ± 2.5	11.0 ± 2.7	10.7 ± 2.6
Stiffness, mean ± SD	4.6 ± 1.0	4.6 ± 0.9	4.6 ± 1.0
Physical function, mean ± SD	41.9 ± 6.4	42.2 ± 10.1	42.0 ± 8.4

*CFA* сetylated fatty acid, *cm* centimeter, *SD* standard deviation, *VAS* visual analog scale, *ROM* range of motion, *WOMAC* Western Ontario and McMaster Universities Osteoarthritis Index, *min* minimum, *max* maximum.

### Patient-reported outcomes (VAS, WOMAC)

**Fig. 2:**
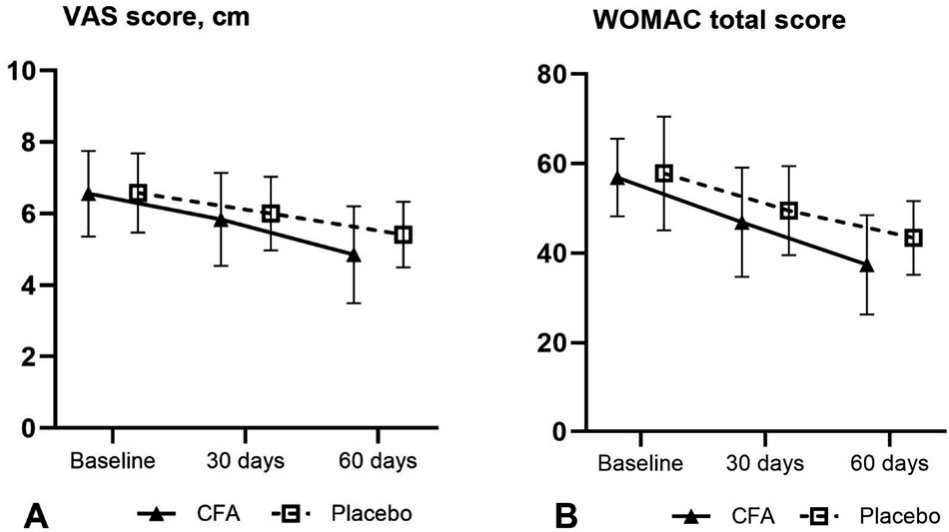
VAS and WOMAC score changes. Mean VAS score **A** and WOMAC total score **B** at different time points.

The additional beneficial effect of incorporating the CFA dietary supplement into the treatment regimen became evident after 60 days of treatment, as shown in Fig. 2 and Table 2. The mean reduction in VAS-measured pain intensity was −1.7 ± 0.8 cm in the CFA group compared to −1.1 ± 0.7 cm in the placebo group, with a statistically significant difference between the groups (*p* < 0.005) (Fig. 2A).

**Table 2:** Change in efficacy parameters (VAS, ROM, WOMAC) from baseline after 60 days of treatment.

Outcome	CFA (*N* = 30) Mean ± SD [95% CI]	Placebo (*N* = 29) Mean ± SD [95% CI]	CFA-Placebo Difference Mean (SE) [95% CI]	*p*-value*
VAS score (cm)	−1.7 ± 0.8 [−2.0, −1.4]	−1.1 ± 0.7 [−1.4, −0.8]	−0.6 (0.2) [−1.0, −0.2]	<0.005
WOMAC Total score	−19.5 ± 8.5 [−22.7, −16.4]	−15.8 ± 9.1 [−19.3, −12.3]	−3.7 (2.3) [−8.3, 0.8]	0.108
ROM (degrees)				
Flexion	3.8 ± 3.2 [2.6, 5.0]	1.3 ± 1.3 [0.8, 1.8]	2.5 (0.6) [1.2, 3.7]	<0.001
Extension	−0.8 ± 1.7 [−1.4, −0.2]	−0.3 ± 0.9 [−0.6, 0.1]	−0.5 (0.4) [−1.2, 0.2]	0.152
External rotation	2.9 ± 2.3 [2.1, 3.8]	1.3 ± 1.1 [0.9, 1.7]	1.6 (0. 5) [0.7, 2.6]	0.001
Internal rotation	1.0 ± 1.1 [0.6, 1.4]	0.5 ± 0.7 [0.2, 0.7]	0.5 (0.2) [0.1, 1.0]	0.032

*CFA* сetylated fatty acid, *cm* centimeter, *CI* confidence interval, *SE* standard error, *SD* standard deviation, *VAS* visual analog scale, *ROM* range of motion, *WOMAC* Western Ontario and McMaster Universities Osteoarthritis Index. * - two-side *t*-test.

The decrease in the WOMAC total score was also more pronounced in the CFA group compared to the placebo group (−19.5 ± 8.5 vs. −15.8 ± 9.1), although this difference was not statistically significant (*p* = 0.108) (Fig. 2B).

However, results from the sensitivity analysis indicated that the outlier factor significantly affected the WOMAC total score. After excluding one outlier, the mean difference between groups was −5.0 (95% CI [−8.9, −1.0]) in favor of CFA, which became statistically significant (*p* = 0.041).

**Fig. 3:**
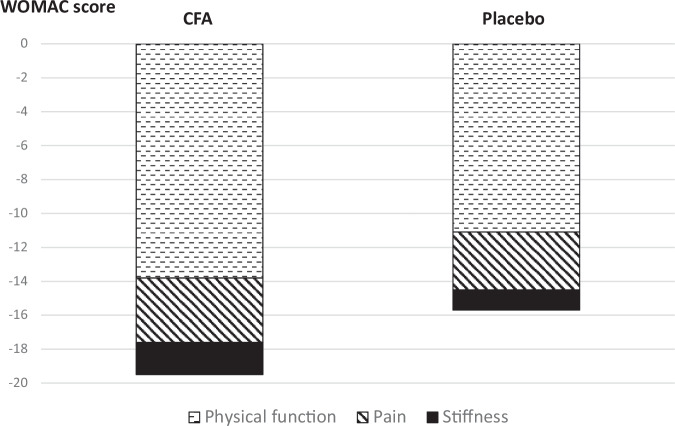
WOMAC score subscale. Contribution of the three WOMAC subscales (pain, stiffness, and physical function) to the change in the total WOMAC score after 60 days of treatment.

Notably, improvement in the physical function subscale was the primary contributor to the differences in WOMAC total score changes between the groups, as illustrated in Fig. 3.

### Instrumental assessment of joint range of motion (ROM)

An improvement in the range of motion of the knee was observed after 60 days of treatment (Table 2). The most pronounced and statistically significant differences in favor of CFA were seen in flexion (*p* < 0.001) and external rotation (*p* = 0.001). Differences in extension and internal rotation were negligible.

Additionally, secondary endpoints assessing instrumental and self-reported parameters after 30 days of treatment were evaluated. The mean differences between the groups in VAS score and in WOMAC total score favored the CFA group but did not reach statistical significance. However, the difference in mean ROM improvement was most pronounced 1.8 (95% CI [1.0, 2.5]) vs. 0.5 (95% CI [−0.1, 1.1]) degrees, and statistically significant in favor of the CFA group for knee flexion (*p* = 0.006), similar to the assessment on Day 60.

### Compliance and adverse events

Adherence to therapy at Visit 2 and Visit 3 was 100% and 100% in the CFA group, and 100% and 96.7% in the Placebo group, respectively.

A total of six treatment-emergent adverse events (nausea, diarrhea, headache, and increased blood pressure) were reported in 4 out of 30 patients (13.3%) during the 60 days of CFA treatment. No patients (0%) in the Placebo group reported any such events. In all cases, the adverse events were mild in severity and were unlikely or possibly related to the investigational product. No patients permanently discontinued the investigational product or were withdrawn from the study due to adverse events.

## Discussion

In the group of musculoskeletal diseases, osteoarthritis is thought to be the most prevalent. Women experience higher osteoarthritis incidence rates than men, particularly after the age of 50. This discrepancy is largely attributed to biological factors, including hormonal differences that may influence cartilage metabolism and joint health [24]. Knee osteoarthritis is more common in women compared to men, in the review of Pereira et al. a prevalence of about 10% more in women than man was found [25].

Our study included a prevalence of women (85%) than men, and the prevalence is higher in the CFA group (90%) then in placebo group (80%). These gender differences do not reflect the prevalence in the general population, and this may influence the response to treatment. Previous studies have reported higher inflammatory activity in knee osteoarthritis women than men [24], but in our study inflammatory markers were not recorded. Assuming, based on previous knowledge, that women might be more inflamed than men, it turns out that the CFA group might be more difficult to treat than the placebo group. Since the treatment was more effective in the CFA group than in the placebo group, this gender imbalance may have made the differences smaller than they might be in the general population.

Various approaches, including different lifestyle and nutritional recommendations [9], HA infiltration [26], topical and oral NSAIDs administration [27], have been explored as an “adjuvant” treatment for alleviating pain and discomfort in OA patients. However, none of them appear to represent the final remedy in solving the pathological or symptomatic aspects of OA.

The findings of our study align with previous research on the efficacy and safety of CFAs in patients with OA and axial discogenic back pain.

In Hesslink et al. study evaluating a formulation with a different CFA mixture, 64 patients with knee osteoarthritis received either a combination of CFAs (six capsules or 2.1 g per day) or a placebo for 68 days. Similar to our study, the CFA group showed a statistically significant improvement in knee flexion, whereas knee extension remained unchanged in both groups [12].

The overall improvement in mobility grades is greater in the Hesslink study than in our study (10.1° vs. 1.8°). It can be assumed that the difference is due to the lower amount of active ingredient (2.1 g vs. 1.5 g per day), or to differences in the sample analyzed, with a higher prevalence of women in our study than in the other one.

One possible explanation is that, in patients with OA, various posterior knee structures contribute to limited extension. These include the posterior capsule and the medial collateral ligament, which may undergo degeneration, fibrosis, and shortening. Additionally, knee flexor muscles such as the popliteus, short head of the biceps femoris, and gastrocnemius may further restrict knee extension [28].

In Pelak et al. study, despite the small sample size (27 patients) and lack of control group, the same CFA-based oral supplement of our study in different dosage (1.2 g per day) demonstrated to be able to reduce pain and disability in patients with axial discogenic low back pain [20].

Topical CFA formulations, applied 2–3 times per day, were also effective in reducing pain and improving functional performance in individuals with knee or hand OA [13, 16, 17, 21]. In Ariani et al. study, an average improvement of 20% in WOMAC total scores in patients with knee OA was observed. The topical CFA formulation was applied 2 times per day for only one week. In our study, a greater improvement was observed in the CFA group (−19.5 mean points from an average of 56.9 points, 34.3%) probably due to the longer intake time. In both studies, results exceeded the minimal clinically important change threshold [29, 30].

Notably, the efficacy of both topical application and oral supplementation of CFA suggests a common effect on joint and tendon structures. The dosage form under investigation (food gel) and its once-daily regimen appear to be the most convenient, as evidenced by the 100% treatment compliance observed in our study. The safety profile of the investigational product resulted favorable, considering that only 4 out of 30 patients reported mild adverse events, and none withdrawn from the study due to adverse events.

Thus, incorporating a CFA oral supplement into the treatment regimen for knee osteoarthritis patients provides superior efficacy in reducing pain severity, as assessed by the VAS scale, and range of motion improvement compared to placebo while maintaining a favorable safety profile. Further research is warranted to assess the long-term efficacy and safety of CFAs in this population.

## Limitation

The study has some limitations: it was conducted in a small group of patients, larger RCTs would generate more data and provide new insights regarding the CFA effectiveness. It could be useful to also investigate the biochemical changes that occur in these patients after taking the food supplement and whether the improvement is maintained over time, also planning an observation period without supplementation. A better assessment of the effect of different dosages, a direct comparison to other administration routes (topical/transdermal) could be also useful to add further information about the treatment efficacy. A further limitation is that this study included a majority of women (85%), thus limiting a complete understanding of what may occur in the general population. Finally, it should be noted that the study was registered retrospectively on clinicaltrials.gov, but the intended study methodology has always been this from the beginning, and none was changed from the original protocol.

## Data Availability

The data used for this research can be available upon reasonable request.
